# Circulating concentrations of GLP-1 are associated with coronary atherosclerosis in humans

**DOI:** 10.1186/1475-2840-12-117

**Published:** 2013-08-16

**Authors:** Katja Piotrowski, Melanie Becker, Julia Zugwurst, Ingeborg Biller-Friedmann, Gerald Spoettl, Martin Greif, Alexander W Leber, Alexander Becker, Rüdiger P Laubender, Corinna Lebherz, Burkhard Goeke, Nikolaus Marx, Klaus G Parhofer, Michael Lehrke

**Affiliations:** 1 Department of Internal Medicine I, University Hospital Aachen, Pauwelsstraße 30, 52074, Aachen, Germany; 2 Department of Internal Medicine II, Ludwig-Maximilians University Munich, Munich, Germany; 3 Department of Internal Medicine I, Ludwig-Maximilians University Munich, Munich, Germany; 4 Institute of Medical Informatics, Biometry and Epidemiology, Ludwig-Maximilians University Munich, Munich, Germany

**Keywords:** GLP-1, Atherosclerosis, Coronary CT angiography

## Abstract

**Background:**

GLP-1 is an incretine hormone which gets secreted from intestinal L-cells in response to nutritional stimuli leading to pancreatic insulin secretion and suppression of glucagon release. GLP-1 further inhibits gastric motility and reduces appetite which in conjunction improves postprandial glucose metabolism. Additional vasoprotective effects have been described for GLP-1 in experimental models. Despite these vasoprotective actions, associations between endogenous levels of GLP-1 and cardiovascular disease have yet not been investigated in humans which was the aim of the present study.

**Methods:**

GLP-1 serum levels were assessed in a cohort of 303 patients receiving coronary CT-angiography due to typical or atypical chest pain.

**Results:**

GLP-1 was found to be positively associated with total coronary plaque burden in a fully adjusted model containing age, sex, BMI, hypertension, diabetes mellitus, smoking, triglycerides, LDL-C (low density lipoprotein cholesterol), hsCRP (high-sensitive C-reactive protein), and eGFR (estimated glomerular filtration rate) (OR: 2.53 (95% CI: 1.12 – 6.08; p = 0.03).

**Conclusion:**

Circulating GLP-1 was found to be positivity associated with coronary atherosclerosis in humans. The clinical relevance of this observation needs further investigations.

## Background

Hyperglycemia and insulin resistance are determinants of cardiovascular risk with fasting blood glucose and insulin concentrations being directly predictive for future cardiovascular events [1,2]. Nevertheless, intensified glucose lowering therapies have failed to substantially lower cardiovascular risk in diabetic patients [3].

Recent reports suggest GLP-1 modulating drugs to hold beneficial cardiovascular characteristics [4]. GLP-1 is an incretine hormone which is secreted from intestinal L-cells in response to nutritional stimuli. Circulating GLP-1 binds to its receptor expressed on pancreatic beta-cells leading to glucose dependent insulin secretion. In addition, GLP-1 suppresses glucagon release from pancreatic alpha cells, impairs gastric motility and reduces appetite which in conjunction improves postprandial glucose metabolism [5]. Furthermore, anti-inflammatory and atheroprotective effects have been described for GLP-1 which extends to cardioprotection during acute myocardial infarction [4]. Relevance of endogenous GLP-1 serum levels for cardiovascular risk prediction in humans has so far not been considered. Impaired GLP-1 secretion has been reported in obese and diabetic subjects in some studies [6-8]. This was however not confirmed in a recent meta-analysis which reported increased GLP-1 serum concentrations in diabetic individuals [9]. Similarly, others have found elevated GLP-1 serum levels in obesity and insulin resistance [10]. This study aimed to assess associations between GLP-1 serum levels and coronary atherosclerosis in humans.

## Methods

Blood samples were collected randomly (independent of fasting) in 303 consecutive patients who underwent DSCT-coronary angiography for exclusion of coronary artery stenosis due to stable typical or atypical chest pain. Detailed information regarding recruitment, baseline characteristics, dual-source multi-slice CT-coronary angiography, dual-source CT image analysis, pericardial fat assessment, and laboratory procedures have previously been published and are summarized in Table 1. Briefly, the coronary tree was segmented according to the suggestions of the AHA into a 15 segment model. Each segment was further divided into a proximal and a distal segment. Each segment was then classified as containing or not containing a plaque. Based on the number of diseased segments a plaque score was calculated. Adequate image quality for evaluation of coronary plaques was obtained in 281 of 303 patients. The study protocol was approved by the Ethics Committee of the Ludwig-Maximilians-University Munich.

**Table 1:** Values are presented as n or median (interquartile range)

**Characteristics**	**n = 303**
**Age (yrs)**	63 (55–70)
**Sex**	
Male	202
Female	101
**Body mass index (kg/m**^**2**^**)**	26.2 (24.1-29.0)
Hypertension^*^	
Yes	147
No	126
Diabetes mellitus^*^	
Yes	20
No	253
**Smoker**^**†**^	
Yes	42
No	230
**Family history of CAD**^**†**^	
Yes	77
No	195
**Laboratory profile**	
LDL-cholesterol (mg/dl)	122 (95–149)
HDL-cholesterol (mg/dl)	52 (44–59)
Triglycerides (mg/dl)	143 (106–207)
High-sensitivity CRP (mg/dl)	0.23 (0.05-0.54)
TNFa (pg/ml)	6,3 (4.5-7.9)
eGFR (ml/min)	71 (63–80)
IL6 (pg/ml)	0 (0–3.2)
GLP-1 (pM)	2.9 (1.5-4.9)
**Medical treatment**^**‡**^	
Statin	120
Aspirin, Plavix or Marcumar	170
Betablocker	173
ACE-I or ARB	138
Diuretics	87
Insulin or OAD	16
**CT data**	
**Pericardial adipose tissue volume (ml)**^**§**^	190 (132–259)
**Number of coronary artery plaques (total)**^**||**^	3 (1–6) (range 0–26)
Number of calcified plaques	1 (0–3) (range 0–22)
Number of mixed plaques	0 (0–1) (range 0–10)

^*^History of diabetes and hypertension is known in 273 patients.

^†^History of smoking and family history of CAD is known in 272 patients.

^‡^Medication is known in 258 patients.

^§^Adequate image quality for evaluation of PAT volume was obtained in 287 patients.

^||^Adequate image quality for evaluation of coronary plaques was obtained in 281 patients.

### Protein chemistry

GLP-1 serum levels were determined using chemiluminescent enzyme-linked immunosorbent assay (ELISA) after ethanol extraction from 300 ul of plasma. Antibodies were purchased from Bioporto (Gentofte, Denmark). The primary antibody was HYB 147–06, which reacts with the amidated C-terminus of GLP-1 (1-36amide), GLP-1 (7-36amide) and GLP-1 (9-36amide). The detection antibody was biotinylated HYB 147–12 which reacts to the mid-molecular epitope of GLP-1. The Intra-Assay and Inter-Assay CV were 7.29% and 12.79%. The assay did provide a linear standard curve (R = 0.98) with the lowest standard being used at 1.5 pM which provided a 10 fold higher signal than the blank sample. Samples were stored at -70˚ with one freeze/thaw cycle performed before GLP-1 measurement. We have found excellent freeze/thaw stability of GLP-1 in human plasma samples. Detection of GLP-1 after 1 to 4 freeze/thaw cycles in probes from 3 different individuals yielded an intra-assay variability of 9,23% which is comparable to the general intra-assay variability. To further validate the assay we performed mixing and spiking experiments. The mixture of equal amounts of two different plasma samples performed for 3 different pairs of samples provided a mean recovery of 93% of the expected GLP-1 concentration. Spiking experiments of 5, 10, 50, 100 and 500 pM standards to a known plasma sample provided a mean recovery of 91% of the expected concentration.

### Statistics

A multiple logistic regression model based on complete cases was used where the effect of GLP1 is adjusted for age, sex, BMI, hypertension, diabetes, smoking, LDL, CRP, GFR and triglycerides. Fractional polynomials were used for evaluating non-linear relationships between continuous covariates and the logarithmic odds for plaques. Further, generalized variance inflation (gVIF) factors were used for evaluating the presence of multicollinearity. A gVIF greater than 4.0 is usually indicates substantial multicollinearity. As a result of the multiple logistic regression model, odds ratios with corresponding 95% confidence intervals and p-values (two-sided) are reported. A p-value of lower than 0.05 is considered statistically significant. To evaluate the relationship between GLP1 and triglyceride and between GLP1 and eGFR, linear regressions based on complete cases with each variable transformed by the natural logarithm were used and the regression coefficients are reported. All statistical analyses were performed by using the statistical software R (version 2.13.1).

## Results

### Levels of total GLP-1 are associated with coronary plaque burden in humans

Serum concentrations of GLP-1 were assessed in a cohort of 303 patients undergoing coronary artery CT angiography due to typical or atypical chest pain. Baseline characteristics of the study have been published previously and are presented in Table 1[11]. Briefly, the mean age of the study population was 63 years, 33% were female, the body mass index was 26.2 kg/m^2^, hypertension was present in 48%, family history of coronary artery disease (CAD) in 25%, 7.2% were smokers and 7% had diabetes. The mean amount of coronary plaque burden detected by CT angiography was 3 (range 0 to 26) with no coronary plaques being detectable in 60 patients. Median GLP-1 in random non-fasting serum samples was 2.9 pM (IQR: 1.5-4.9). Using spearman association GLP-1 (log scale) was found to positively correlate with serum triglycerides (log scale) (Rho: 0.27; p < 0.0001), while a weak negative correlation was found with kidney function as defined by eGFR (estimate glomerular filtration rate; log scale) (RHO: -0.14; p < 0.05). Additional regression beta analysis for these two parameters found a statistically significant relationship between GLP-1 and triglycerides (regression coefficient: 0.35 (95% confidence interval: 0.18-0.52)) and a near-significant relationship between GLP1 and eGFR (regression coefficient: -0.38 (95% confidence interval: -0.77-0.01)). No obvious correlation was detected with other cardiovascular risk factors in the total cohort.

Within the framework of the multiple logistic regression model, non-linear relationships were detected for GLP1, CRP, triglyceride and GFR. For these four variables each non-linearity is best modeled by taking the natural logarithm of GLP1, CRP, triglycerides and eGFR. Besides, the maximum of the observed generalized variance inflation factors was 2.08 for age which indicates the absence of serious multicollinearity problems. The multiple logistic regression model revealed that the logarithm of GLP-1 was significantly associated with total coronary plaque burden in a model adjusted for age, sex, BMI, hypertension, diabetes, smoking, LDL, log. CRP, log. eGFR and log. triglycerides (OR: 2.53; 95% CI: 1.12-6.08; p = 0.03) (Table 2). The unadjusted OR for GLP-1 with total coronary plaque burden was 2.12 (95% CI: 1.15-4.08; p = 0.02).

**Table 2:** Multivariate Association of GLP-1 and relevant cardiovascular risk factors with total plaque burden as assessed by CT-angiography

**Variables**	**Total plaque**	
	**OR (CI)**	***p***
log GLP-1	2.53 (1.12 – 6.08)	**0.03**
Age	1.15 (1.10 – 1.22)	**< 0.001**
Sex	13.92 (4.47 – 51.53)	**< 0.001**
BMI	1.20 (1.05 – 1.39)	**0.01**
Hypertension	1.01 (0.38 – 2.65)	0.98
Diabetes	2.11 (0.47 – 12.42)	0.36
Smoking	6.04 (1.37 – 35.42)	**0.03**
LDL	1.00 (0.99 – 1.01)	0.80
log CRP	1.15 (0.45 – 3.18)	0.78
log TG	2.22 (0.71 – 7.41)	0.18
log eGFR	1.15 (0.08 – 18.37)	0.92

Odds ratio and 95% CI increase in plaque quantity for 1 pM of total GLP-1 raise in plasma levels are presented.

*Abbreviations:LDL* (low density cholesterol), *CRP* (hsCRP), *TG* (triglyceride), *eGFR* (estimate glomerular filtration rate), *log*. Logarithmic.

Bold numbers indicate significant associations with p < 0.05.

## Discussion

In this study we found a positive association between GLP-1 serum levels and coronary artery disease in humans. This observation seems surprising taking the vasoprotective efficacy described for GLP-1 and its analogues in experimental models which seem to be confirmed by early clinical observations [4,12]. Furthermore, inhibition of DPP-4 dependent degradation of GLP-1 was found to reduce atherogenesis and increase plaque stability in mice while preliminary clinical data suggest similar vasoprotective efficacy in humans [13,14]. Nevertheless, others have reported GLP-1 serum levels to be increased in patients with high cardiovascular risk due to accumulation of components of the metabolic syndrome and type 2 diabetic subjects [9,10]. Our study confirms the previously described positive association between GLP-1 serum levels and serum triglycerides [10]. Increased GLP-1 concentrations in states of metabolic risk like hypertriglyceridemia could represent a counter regulation to increase insulin secretion in states of insulin resistance. Consistently, GLP-1 serum concentrations were recently found to be increased in type 2 diabetic patients [9]. Still long term treatment with GLP-1 analogues significantly improved glycemic control and produced meaningful weight loss as a major cardiovascular risk factor in type 2 diabetic patients [15,16]. In addition, GLP-1 analogues were found to be non-inferior to metformin treatment in respect to circulating markers of inflammation, oxidative stress and vascular function in a 3 month lasting clinical trail [17] while protective effects of GLP-1 analogues were found in hepatocytes under ischemic stress [18].

GLP-1 is produced from intestinal L-cells from the epithelial layer of the gut mucosa in response to food ingestion, parasympatic nervous activation and endocrine mediators [5]. In addition IL6 as an inflammatory cytokine was recently found to increase GLP-1 secretion under in vitro and in vivo conditions [19]. Coronary atherosclerosis is a chronic inflammatory disease process which has been associated with IL6 [20]. We were however unable to detect associations between GLP-1 and different mediators of inflammation including IL6 in our population, making this an unlikely explanation.

Elimination of GLP-1 primarily depends on the kidney with increased circulating concentrations of GLP-1 being found in patients with chronic kidney disease and renal failure [21,22]. Consistently we found weak negative associations of GLP-1 with kidney function in our population. Chronic kidney disease is a major cardiovascular risk factor. Nevertheless associations of GLP-1 and atherosclerotic plaque burden remained significant after adjustment for kidney function, making impaired GLP-1 elimination in states of renal disease an unlikely explanation for the observed association of GLP-1 with coronary artery disease.

### Study limitations

Our study holds several strength and limitations. This is the first study to establish a link between circulating serum concentrations of GLP-1 and coronary artery disease. This association remained significant in a fully adjusted model after consideration of age, sex, BMI, hypertension, diabetes mellitus, smoking, triglycerides, LDL-C, hsCRP, and eGFR. However as this was an association study, this does not establish a causal relationship between GLP-1 and coronary artery disease. Consequently, clinical implications of this observation still need to be established. Experimental data suggest vasoprotective efficacy of GLP-1, GLP-1 analoga and DPP-4 inhibition [4] which are supported by preliminary clinical data [12,13]. The observed increase of GLP-1 in context of vascular disease might therefore represent a counter regulatory attempt to limit disease progression. Increased GLP-1 secretion in states of metabolic disease might however also contribute to hyperinsulinemia which has been linked with vascular disease [2].

The GLP-1 assay used in this study does detect active GLP-1 in addition to processed metabolites of GLP-1. These GLP-1 metabolites might hold own vascular actions which seem to be mediated independent of the GLP-1 receptor [23]. Additional studies are needed to further discriminate associations between active GLP-1and its metabolites with cardiovascular disease. Furthermore, systemic and coronary artery concentrations of active GLP-1 might differ due to myocardial DPP-4 activity.

Serum samples in our study were collected randomly independent of the last food intake. As food ingestion is a strong stimulus of GLP-1 secretion this might have affected the observed associations [6]. Additional studies are needed to establish whether a similar link can be found under fasting conditions. Non-fasting conditions might however hold predictive relevance for some metabolic biomarkers. This has earlier been reported for serum triglycerides which better predicted cardiovascular risk when assessed in a non-fasting state [24].

Finally, our study results may only be applicable to non-diabetic patients as diabetic patients were clearly underrepresented in the present study (7% of the patients).

Additional investigations in larger cohorts are needed to further explore the association between GLP-1 and cardiovascular disease in larger cohorts under consideration of clinical outcome data.

In summary we found a surprising association of circulating GLP-1 with coronary atherosclerosis in humans. This might represent a contra-regulatory response in states of increased metabolic risk which warrants further investigations.

## Abbreviations

ACE-I: Angiotensin-converting enzyme inhibitors; AHA: American Heart Association; ARB: Angiotensin II receptor blocker; BMI: Body mass index; CAD: Coronary artery disease; DPP-4: Dipeptidyl peptidase-4; eGFR: Estimate glomerular filtration rate; GLP-1: Glucagon-like peptide 1; HDL-C: High density lipoprotein cholesterol; hsCRP: High-sensitive C-reactive protein; IL6: Interleukin 6; LDL-C: Low density lipoprotein cholesterol; PAT: Pericardial adipose tissue; TNFα: Tumor necrosis factor alpha.

## Competing interests

The authors declare that they have no competing interests.

## Authors’ contributions

KP: sample analysis and primary drafting of the manuscript; MB, JZ, IBF and GS: sample analysis; MG, AWL and AB: sample collection and CT analysis; RPL: statistical analysis; CL: sample collection and manuscript drafting; BG, NM and KGP: manuscript drafting; ML: study design and analysis, final manuscript drafting. All authors read and approved the final manuscript.
